# An Image-Based Benchmark Dataset and a Novel Object Detector for Water Surface Object Detection

**DOI:** 10.3389/fnbot.2021.723336

**Published:** 2021-09-24

**Authors:** Zhiguo Zhou, Jiaen Sun, Jiabao Yu, Kaiyuan Liu, Junwei Duan, Long Chen, C. L. Philip Chen

**Affiliations:** ^1^School of Information and Electronics, Beijing Institute of Technology, Beijing, China; ^2^College of Information Science and Technology, Jinan University, Guangzhou, China; ^3^Faculty of Science and Technology, University of Macau, Taipa, Macau, SAR China; ^4^School of Computer Science and Engineering, South China University of Technology, Guangzhou, China

**Keywords:** surface object detection, dataset, detector, baseline, cross-dataset validation

## Abstract

Water surface object detection is one of the most significant tasks in autonomous driving and water surface vision applications. To date, existing public large-scale datasets collected from websites do not focus on specific scenarios. As a characteristic of these datasets, the quantity of the images and instances is also still at a low level. To accelerate the development of water surface autonomous driving, this paper proposes a large-scale, high-quality annotated benchmark dataset, named Water Surface Object Detection Dataset (WSODD), to benchmark different water surface object detection algorithms. The proposed dataset consists of 7,467 water surface images in different water environments, climate conditions, and shooting times. In addition, the dataset comprises a total of 14 common object categories and 21,911 instances. Simultaneously, more specific scenarios are focused on in WSODD. In order to find a straightforward architecture to provide good performance on WSODD, a new object detector, named CRB-Net, is proposed to serve as a baseline. In experiments, CRB-Net was compared with 16 state-of-the-art object detection methods and outperformed all of them in terms of detection precision. In this paper, we further discuss the effect of the dataset diversity (e.g., instance size, lighting conditions), training set size, and dataset details (e.g., method of categorization). Cross-dataset validation shows that WSODD significantly outperforms other relevant datasets and that the adaptability of CRB-Net is excellent.

## Introduction

Water surface object detection plays an increasingly significant role in the areas of autonomous driving such as unmanned surface vehicles (USVs) and water surface vision applications. To detect visual objects more accurately, annotated benchmark datasets (Everingham et al., 2010) are used to validate the different object detection methods, which can avoid the time-consuming process of building their own datasets. According to different object detection methods, a persuasive performance comparison can be presented based on the same annotated benchmark dataset. Nevertheless, there is a dearth of image-based datasets that focus on the application of water surface object detection. Moreover, current water surface datasets still have several drawbacks. For example, the primary issues existing in the boat-types-recognition dataset (Clorichel, 2018) are small data scales, a limited number of surface object categories, and only one climate type. In addition, for large generic image-based datasets, such as MS COCO (Lin et al., 2014), ImageNet (Krizhevsky et al., 2012), and Places 2 (Zhou et al., 2015), the images of water surface visual objects are collected from websites, and there are not enough images for training the different neural networks. Therefore, performance is an issue when a water surface detector is trained on these types of datasets. To address all these issues, it is necessary to build a new water surface dataset with a broad range of water environments, complete categories of common obstacles, multiple climate conditions, and various shooting times.

This paper proposes a novel benchmark dataset called WSODD that has more instances and categories for the detection of common obstacles on water surfaces. It consists of 7,467 water surface images taken by a Hikvision industrial camera, and the resolution of each image is 1,920 ^*^ 1,080. A wide range of environments, such as oceans, lakes, and rivers, are included, and the images in WSODD are obtained under three different shooting time periods (daytime, twilight, and night) and three different climatic conditions (sunny, cloudy, and foggy days). There are 14 categories and 21,911 instances in the proposed fully annotated dataset, with each instance marked by an axis-symmetric bounding box. All of the annotations and original images will be public, and an online benchmark will be set up.

To delve into WSODD, CRB-Net is proposed to serve as a baseline. Water Surface Object Detection Dataset (WSODD) contains many small objects as well as objects that are not easily detected, so the detector extracts deeper semantic features and uses SPP (He et al., 2015) to enhance the receptive field. While fusing cross-scale features, most previous structures are simply stack inputs without distinction. However, these features are at different resolutions, and their contributions to the fused features are often not equal. To solve this problem, we introduce an improved BIFPN (Tan et al., 2020) that can carry out adaptive weight adjustment during feature fusion by attention mechanism and Mish activation (Misra, 2019). Moreover, CRB-Net optimizes the initial value of the anchor frame based on the K-means algorithm, which makes the anchors match the shape characteristics of an obstacle. The main contributions of this paper are:

1. Water Surface Object Detection Dataset, a novel image-based benchmark dataset for water surface object detection, is proposed with the most categories of common obstacles, and the broadest ranges of water environments and weather conditions. The images in WSODD can reflect real visual objects more accurately.
2. A novel detector (CRB-Net) is proposed, and a benchmark of performance comparison with 16 state-of-the-art object detection methods is presented. The results reveal CRB-Net outperforms other methods in terms of detection precision. In addition, we explore the detection performance of various detectors for objects of different sizes in WSODD.
3. A boat-types-recognition dataset is chosen to perform cross-dataset generalization because it is the only publicly available image-based water surface dataset. The results suggest that WSODD has more patterns and properties than boat-types-recognition, and that CRB-Net has excellent generalization ability.

In addition to advancing object detection studies in water surface vision, WSODD will put forth new questions about methods that are worth exploring in the field of machine vision.

## Related Works

### Datasets

Currently, there are not so many datasets for water surface object detection. Boat-types-recognition dataset is the only public image-based dataset which can be found in this area. It contains 1,462 images of the water surface, with three categories of common objects: boat (gondola, inflatable boat, kayak, paper boat, sail boat), ship (cruise ship, ferry boat, freight boat), and buoy. Though the water environments and shooting times of this dataset are significantly abundant, the annotations for object detection are not provided in the dataset.

The generic image-based datasets can also be used for water surface detection. For instance, MS COCO is a large generic dataset, including 91 categories of objects, and a total of 328,000 images. However, there is only one category (boat) related to water surface detection, which contains 3,146 images. Obviously, the number of obstacles and images in this dataset is not enough to assure the effective training of a deep learning neural network. Another dataset named ImageNet provides a large-scale of annotations, but the categories related to water surface object detection include only four kinds: catamaran, trimaran, container-ship and aircraft-carrier, and these images are quite different from the real water surface conditions. Additionally, Places2 is a generic dataset which contains 365 categories, but only five categories are related to water surface, which are harbor, lake, loading-dock, water and river, respectively. Generally speaking, most of these images cannot be used for water surface object detection tasks due to the lack of water surface obstacles. Table 1 shows a comparison of WSODD and other image-based WSODD.

**Table 1:** Comparison of WSODD and other image-based water surface object detection datasets.

**Dataset**	**Dataset's type**	**Main categories**	**Main environments**	**Weather conditions**	**Shooting time**	**Images**
MSCOCO	Generic	1	Sea	Sunny	Daytime	3,146
			Lake	Cloudy		
ImageNet		4	Sea	Sunny	Daytime	1,996
			Lake	Foggy		
Places2		5	Lake	Sunny	Daytime	6,514
			River			
Boat-types-recognition	Specialized	3	Sea	Sunny	Daytime	1,462
			Lake		Twilight	
			River		Night	
WSODD		14	Sea	Sunny	Daytime	7,467
			Lake	Foggy	Twilight	
			River	Cloudy	Night	

*The main categories refer to the number of categories related to water surface object detection. The boat-types-recognition dataset includes many kinds of boats, which are all considered as boat here. Similarly. Images in this Table refer to the number of images related to water surface object detection*.

In addition, there are some video-based WSODDs, such as Singapore-maritime dataset (Prasad et al., 2017), MODD dataset (Kristan et al., 2016), and Visual-Inertial-Canoe dataset (Miller et al., 2018), but most of them also have the problems of little obstacle categories and relatively simple environment, thus it is difficult to achieve better performance of object detection.

### Methods

It is well-known that for early generic object detection methods [e.g., LBP (Ojala et al., 2002), DPM (Felzenszwalb et al., 2010)] it is difficult to extract features from images. Additionally, the precision and speed of object detection are also not satisfied. After 2012, with the development of deep learning, many high-efficiency CNN-based detectors have emerged, which can be mainly divided into two categories: two-stage object detection methods and one-stage object detection methods (Liang et al., 2020). The most famous two-stage object detection method is the R-CNN (Girshick et al., 2014) series [e.g., Faster R-CNN (Ren et al., 2016), Mask R-CNN (He et al., 2017), and Cascade R-CNN (Cai and Vasconcelos, 2018)]. In terms of one-stage object detection method, Yolo (Redmon and Farhadi, 2018) and SSD (Liu et al., 2016) are the most remarkable methods. Moreover, the one-stage detector can also be transformed into an anchor-free detector such as CenterNet (Duan et al., 2019).

As an important part of computer vision, water surface target detection has attracted much attention. Before the emergence of deep learning methods, the method of combining wavelet transform and image morphology (Yang et al., 2004) is the dominant method to realize the water surface object detection. An object detection system was introduced (Wijnhoven et al., 2010) based on HOG (Dalal and Triggs, 2005) for finding ships in the maritime video. Matsumoto (Matsumoto, 2013) proposed a HOG-SVM method to detect ships on the images from ship mounted camera. In 2016, Kaido et al. used support vector machine and edge detection in the detecting of ships. Moreover, a vessel number plate identification was proposed by using two cameras and identification of various vessels passing through the port (Kaido et al., 2016).

The technique of deep learning significantly pushes the progress of this field. Due to variation in size, appearance, and disturbances, unsupervised methods (Liu et al., 2014) are severely limited. Therefore, it is more common to use supervised methods (Mizuho et al., 2021). Yang (Yang et al., 2017) proposed an architecture which uses Fast R-CNN to realize the identification and classification of ships. In addition, a hybrid ship detection method (Yao et al., 2017) was presented that integrates deep learning methods. Specifically, they utilized Deep Neural Networks (DNNs) and Region Proposal Networks (RPNs) to obtain a 2D bounding box of target ships. Furthermore, a fast detection method was designed for surface objects based on ResNet (Chae et al., 2017), and the speed of object detection can reach 32.4 frames per second (FPS). Moreover, Qin (Qin and Zhang, 2018) adopted FCN for surface obstacle detection, which has a good robustness. In 2019, an improved RBox-based water surface target detection framework (An et al., 2019) was proposed to obtain accuracy recall rate and precision of the detection. And Sr et al. proposed a ship algorithm using an improved YOLO and multi-feature ship detection method to detect ships. For this method the SIFT features are reduced by MDS (multi-dimensional scaling) and RANSAC (random sample consensus) was used to optimize SIFT feature matching and effectively eliminate mismatching (Sr et al., 2019). Moreover, a real-time water surface object detection method (Zhang et al., 2019) was proposed based on improved Faster R-CNN, which includes two modules and integrates low-level features with high-level features to improve detection accuracy. The proposed method was utilized to detect the floats on the water surface via a three-day video surveillance stream of the North Canal in Beijing, and validated its performance. In addition, the deep residual network and cross layer jump connection policy was employed (Liu et al., 2019) to extract the advanced ship features which help improve the performance of object recognition. In 2020, a method was proposed based on yolov2 (Chen et al., 2020b) to detect small ship, and which can also be utilized for identification of various obstacles on the water surface. And H-Yolo (Tang et al., 2020) was proposed to detect ship based on region of interest preselected network. The principle of this approach is to distinguish suspected areas from the images based on hue, saturation, value (HSV) differences between ships and the background. Then, a water surface detection method was proposed called Yolov3-2SMA (Li et al., 2020), allowing real-time and high-precision object detection in dynamic aquatic environments. Moreover, Jie et al. (2021) improved yolov3 to detect ships in inland waterways, the mAP and FPS of the improved method increased by about 5 and 2%. Recently, ShipYolo (Han et al., 2021) was introduced to solve the problem of missed inspection of small-scale ships. This algorithm designed a new amplified receptive field module with dilated convolution and Max-Pooling, which improves the model's acquisition of spatial information and robustness of target space displacement. However, most of the above methods are not feasible to be applied by autonomous ships are based on the static cameras for port management and thus do not match the shipborne surveillance systems on moved autonomous ships (Jie et al., 2021). Furthermore, even with all the proposed algorithms, they still encountered drawbacks of efficiency and accuracy.

## Benchmark Dataset for Water Surface Object Detection

Most researchers believe that a dataset should cover as many real images as possible and have as little personal bias as possible in the annotation process. The dataset proposed in this paper includes more instances, categories, environments, shooting times, and weather conditions than previous datasets.

### Image Acquisition

All of the images in the WSODD were captured by an industrial 4G HD camera during the period of July 16 to September 10, 2020. The temperature range was 20–35°C.

In order to enrich the environments and reflect the real world as accurately as possible, five water areas consisting of three types of water surface environments were chosen. These are the Bohai Sea (Dalian, Liaoning Province, China; ocean), the Yellow Sea (Yantai, Shandong Province, China; ocean), Xuanwu Lake (Nanjing, Jiangsu Province, China; lake), Nanhaizi Lake (Beijing, China; lake), and the Yangtze River (Nanjing, Jiangsu Province, China; river).

For the purpose of enriching the climate categories, every water environment was photographed under different weather conditions, such as sunshine, clouds, and fog.

At the same time, the obstacles were photographed under different lighting conditions including midday (high light), dusk (low light), and evening (very low light), so that enough research materials were collected for the dataset.

**Figure 1:**
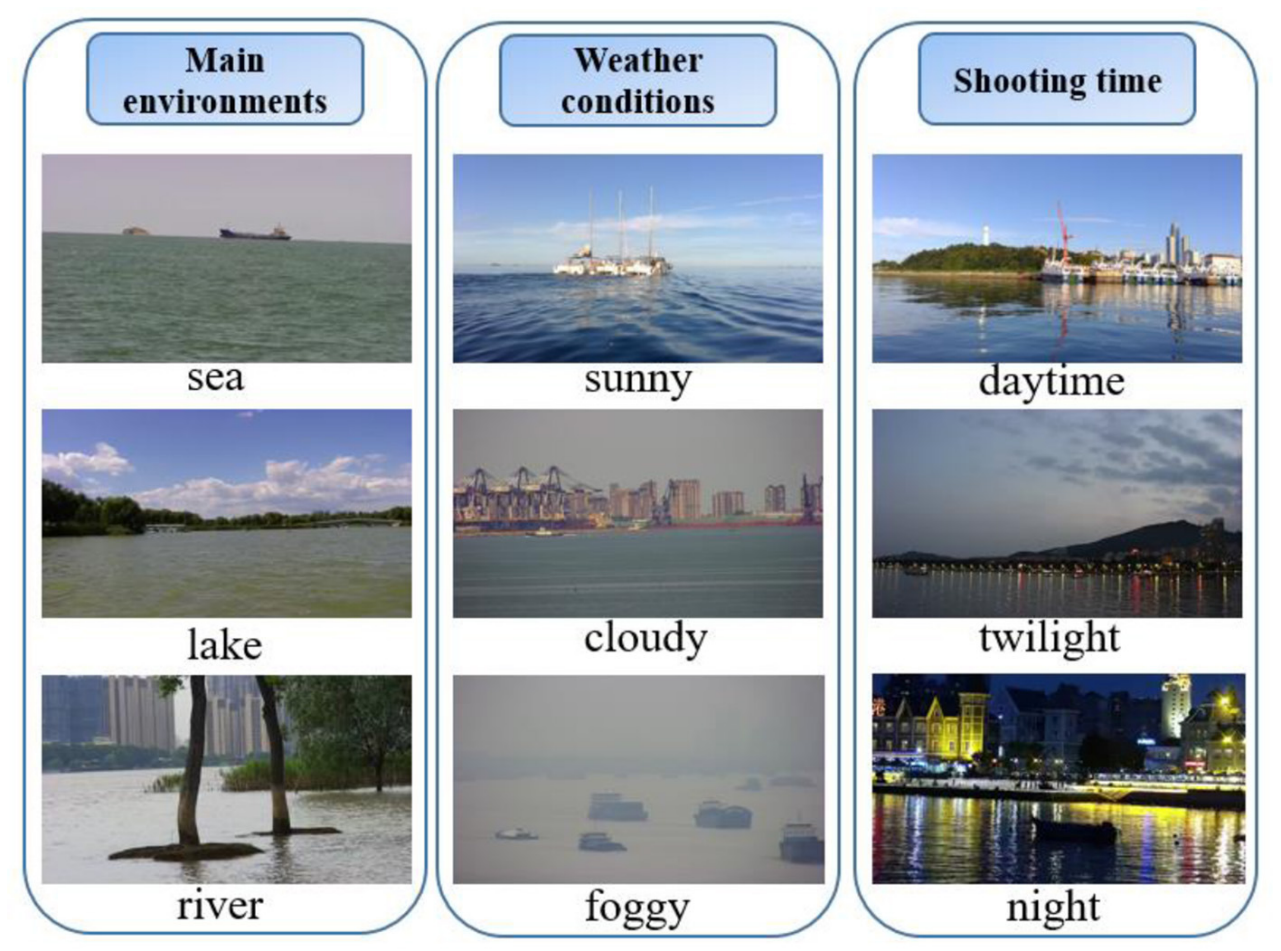
Typical environments and conditions in WSODD.

Figure 1 shows some typical environments of WSODD. It is clear that the images not only show numerous surface obstacle information, but also include relevant information about the surrounding sea, land, and port, which is closer to the actual water surface object detection application (Kristan et al., 2014).

### Selection of Categories

**Figure 2:**
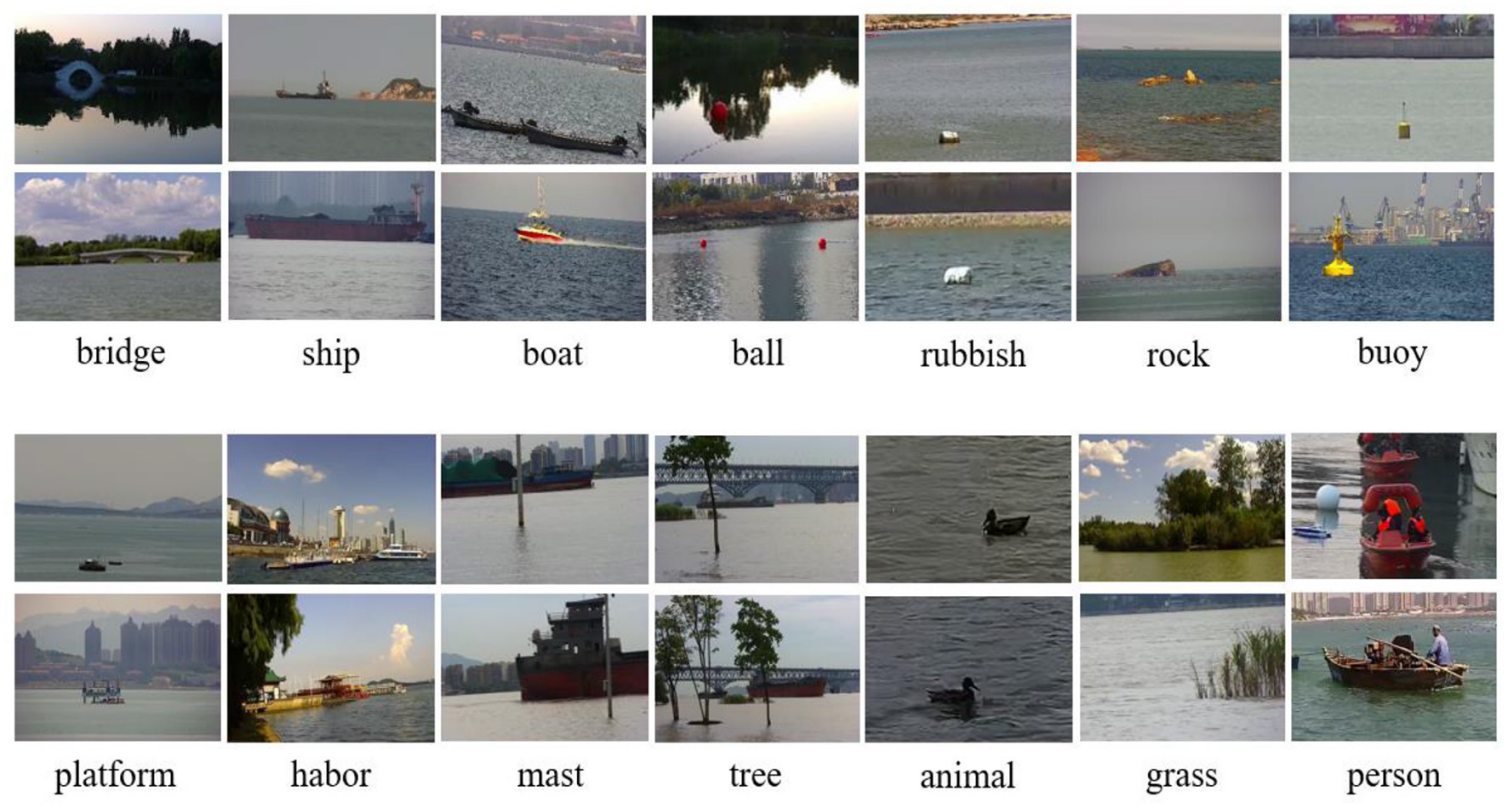
Typical categories in WSODD.

Water Surface Object Detection Dataset was selected and annotated with 14 common objects on the surface, namely, boat, ship, ball, bridge, rock, person, rubbish, mast, buoy, platform, harbor, tree, grass, and animal. Figure 2 displays two images of each category.

The core criterion for choosing the objects is their commonness in real water environments. Water Surface Object Detection Dataset's object category division is relatively broad. For example, the ship category includes large warships and passenger ships; at the same time, other researchers can test methods directly based on this dataset, or classify an existing category in more detail. Table 2 lists the number of images and instances for each category of WSODD.

**Table 2:** Images and instances for each category.

**Label**	**Images**	**Instances**
Boat	4,325	8,179
Ship	1,832	3,423
Ball	652	2,609
Bridge	1,827	2,014
Rock	696	1,540
Person	357	695
Rubbish	461	669
Mast	177	354
Buoy	153	167
Platform	480	614
Harbor	1,211	1,224
Tree	72	219
Grass	103	110
Animal	50	94
**Total**	**7,467**	**21,911**

### Image Annotation

Water Surface Object Detection Dataset was annotated in two ways, the same as PASCAL VOC (Everingham et al., 2010) and MS COCO (Lin et al., 2014). The annotation files were saved in XML format.

Considering that many researchers conduct experiments based on COCO format annotation files, we will provide the code that can convert VOC files into COCO files. When other researchers want to use COCO format annotation files, they can use this code to easily convert the format.

It is worth noting that this research focuses on annotating a water surface dataset, and does not include land objects. All of the annotations, including the omitted objects, were checked by an engineer in order to ensure more detailed annotation.

### Dataset Statistics

**Figure 3:**
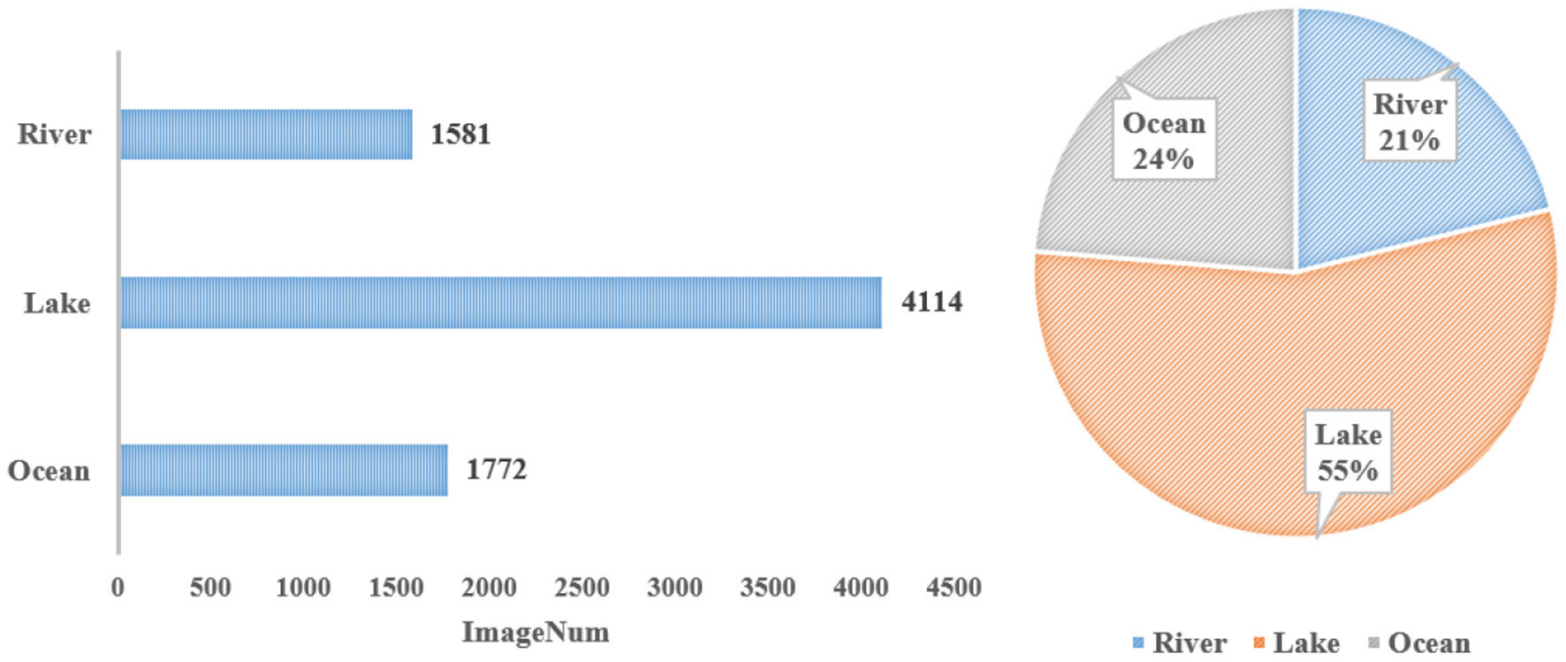
Statistics under different surface environments.

The statistics for different water surface environments are shown in Figure 3. There are 1,771 images of ocean environments, 4,114 images of lake environments, and 1,581 images of river environments, respectively accounting for 24, 55, and 21% of WSODD (Zhang et al., 2020). It should be noted that the ship category in WSODD covers only seas and rivers, because Xuanwu Lake and Nanhaizi Lake are small lakes that cannot host large ships. The platform category only exists for the sea. During the shooting, we found that there are many such platforms in the offshore waters for marine aquaculture and seawater quality testing, but no such objects were found on rivers or lakes. Conversely, the grass classification only exists for rivers and lakes, but not the sea, where no large area of grass has ever been found. A possible reason for this is that the impact of waves will devastate the growth of the grass.

**Figure 4:**
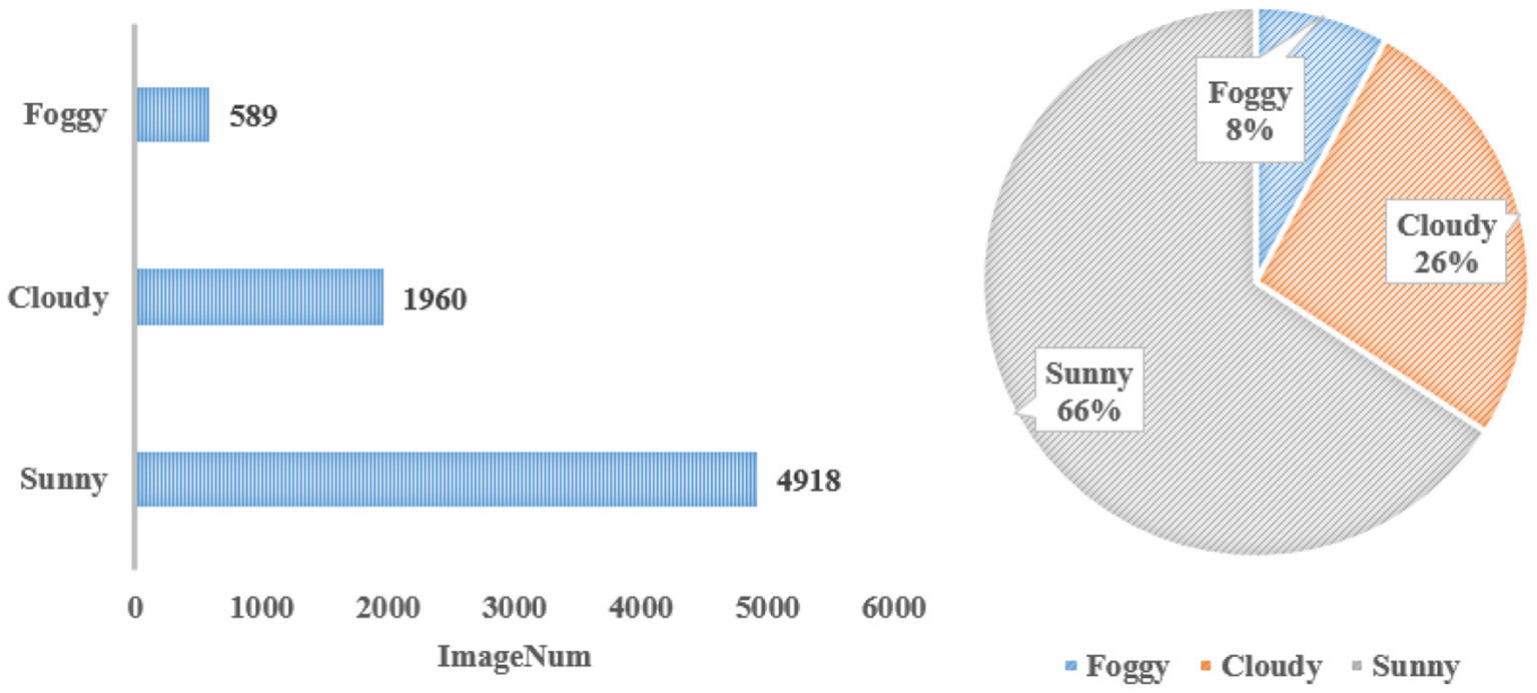
Statistics under different climate conditions.

Figure 4 depicts the number of images under different climatic conditions. The majority of images, 4,918, representing 66% of WSODD, were photographed on sunny days, while the fewest images, 589, or 8% of the dataset, were taken on foggy days.

**Figure 5:**
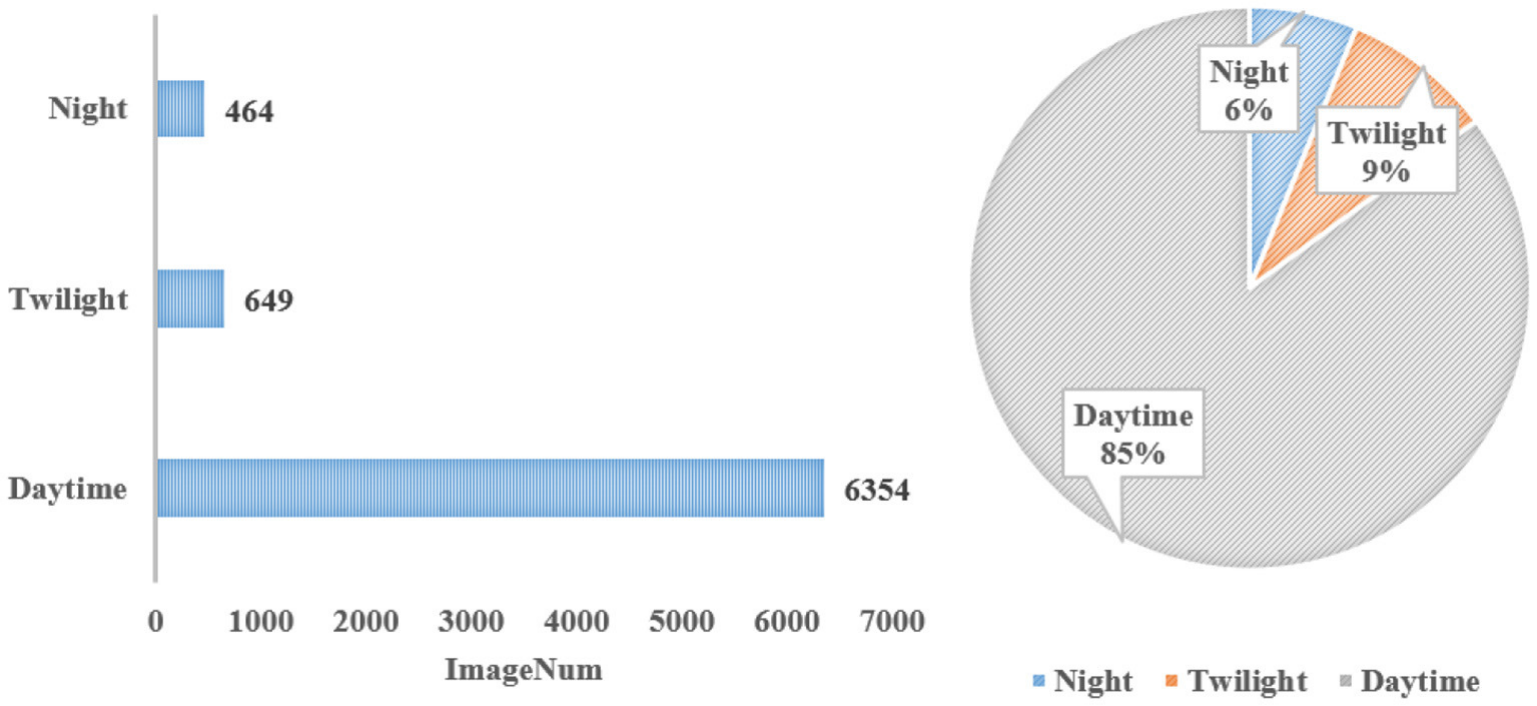
Statistics under different shooting times.

The data for the different shooting times are depicted in Figure 5. The majority of the images, 6,354, or about 85% of the total, were collected in the daytime. In addition, an average of 3.15 instances for each image were taken during the daytime. A similar number of instances were taken for each image at twilight, 3.24 (Alessandro et al., 2018). However, the average number of instances for each image taken at night was 1.19. There are two main reasons for this large discrepancy. One is that the number of objects that continue to move at night, such as boats, is small. The other is that the light is so dim at night that many existing objects cannot be found, especially for objects that are far away from the shooting location or objects that are small.

**Figure 6:**
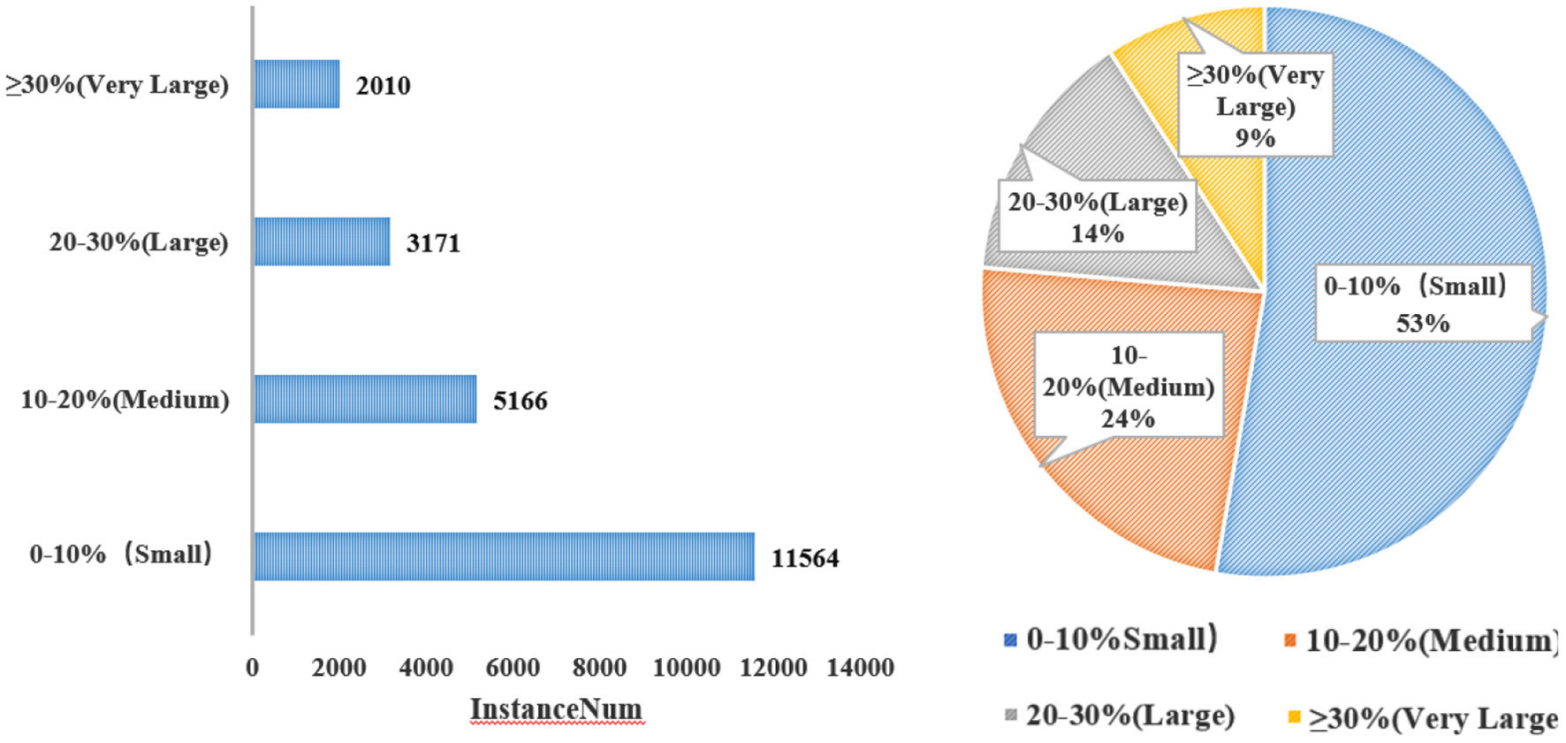
Statistics of the scale of an instance in its image.

Some instances may be as small as 0.01% of an image, while others can be more than 40%. The distinct differences between instances make the detection task even more challenging, because the model must be flexible enough in order to deal with extremely small as well as extremely large objects (Li et al., 2018). Figure 6 depicts the statistics of the scale of an instance in its image.

## Novel Detector for Water Surface Objects

To build the baseline method of WSODD, we proposed CRB-Net, which is an enhanced target detector based on CSPResNet (Wang et al., 2019).

### Network Architecture

**Figure 7:**
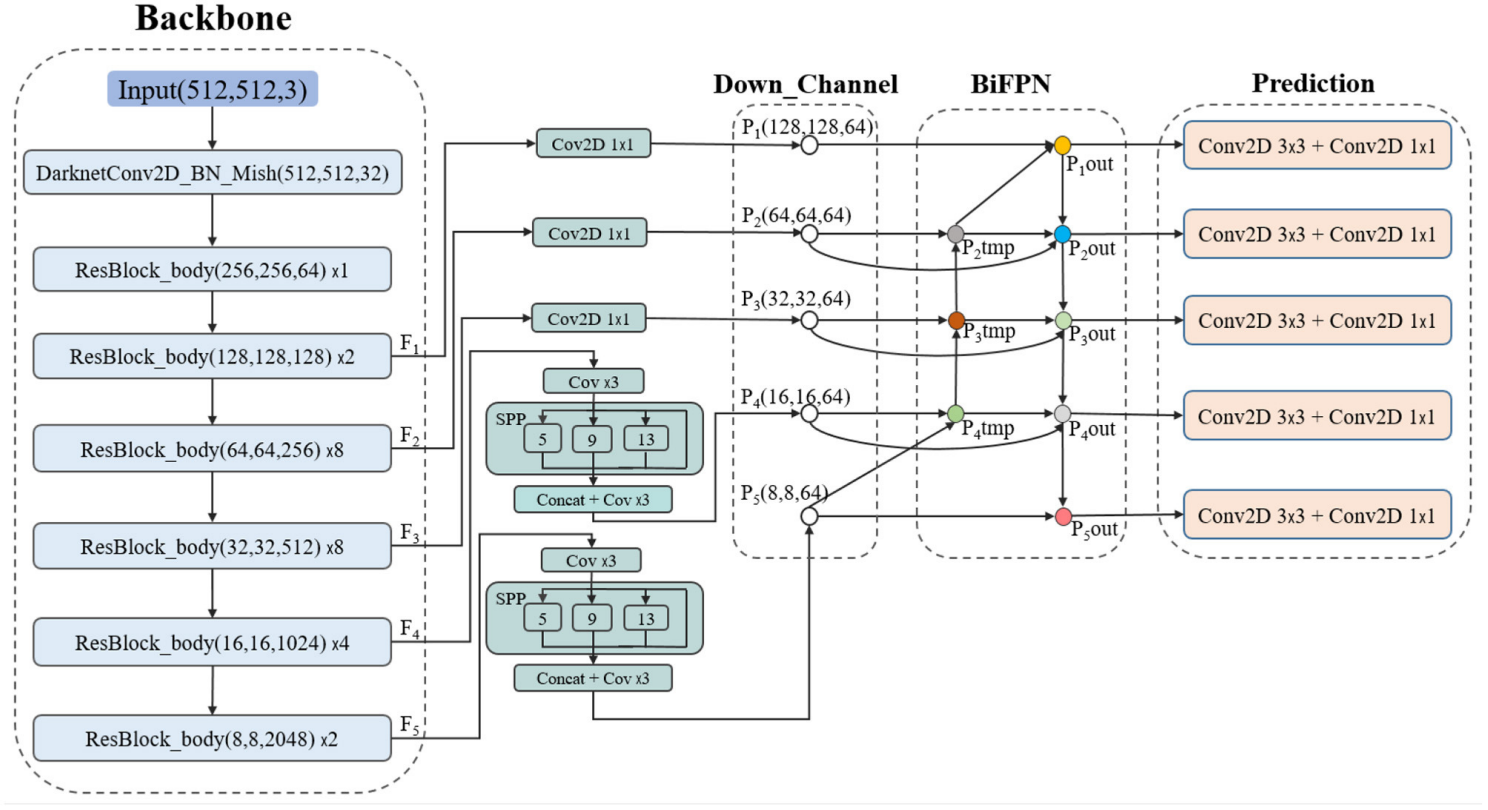
The architecture of CRB-Net.

Figure 7 displays the architecture of CRB-Net. The backbone of CRB-Net uses the ResBlock_building block to obtain five output feature layers, and the feature point in every feature layer is set to three anchors.

In addition, each detection frame of each detection layer was offset based on a different anchor frame. The width and height values of each anchor need to be obtained based on the shape characteristics of the objects to be detected. We use the K-means clustering algorithm to optimize the initial value of the anchor frame, which can make anchors more suitable for water surface scenarios while reducing the training time significantly.

Next, two SPPNets (He et al., 2014) were used to increase the receptive field of F_4_ and F_5_, which can isolate the most significant contextual features.

A common way to fuse features with different resolutions is to resize their resolutions to be the same before adding them up. However, different inputs contribute unequally to the fusion process. To solve this problem, we designed an improved BIFPN by incorporating an attention mechanism.

Finally, the feature layers after semantic fusion were sent into five Yolo heads to obtain the prediction result.

### Network Module Details

#### ResBlock_Body

**Figure 8:**
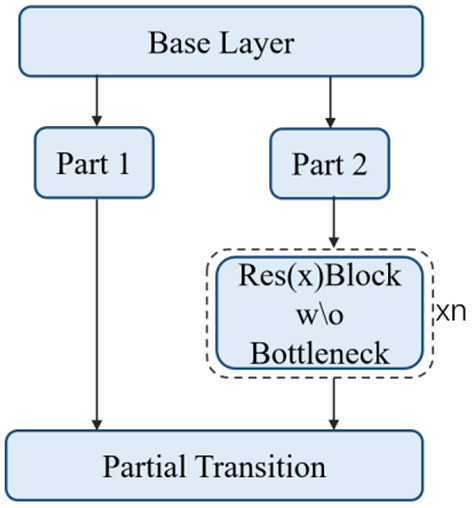
CSPResNet.

This is actually a CSPResNet, whose structure is shown in Figure 8. The residual blocks are stacked in the trunk part. The other part, the residual edge, is connected directly to the end after some processing. This structure alleviates the gradient disappearance problem caused by increasing the depth in the DNN.

#### K-Means Algorithm

To find the optimal clustering effect, we selected multiple groups of different numbers of clusters for experimental comparison. We found that when the number of clusters reached 15, the increase in avg IoU almost stopped (the calculation method of avg IoU was done by calculating IoU for each training set label and the center obtained by clustering, taking the largest IoU value as the value of this label, and finally averaging all of the label values to obtain it). Considering that the risk of model overfitting will increase as the number of clusters increases, 15 cluster centers were finally selected.

#### Improved BIFPN

This integrates the bidirectional cross-scale connections and the fast normalized fusion. The best value of 1.35 was selected as the BiFPN width scaling factor. To better illustrate the fusion process, P2 is chosen as an example to describe the fused features.


(1) $$\begin{array}{r} P_{\text{2}}^{tmp}=Conv\left(\frac{\omega_{1}▪P_{2}^{in}+\omega_{2}▪Resize\left(P_{3}^{in}\right)}{\omega_{1}\text{+}\omega_{\text{2}}\text{+}\beta }\right) \end{array}$$

(2) $$P_{2}^{out}=Conv\left(\frac{\omega_{1}{}^{\prime }▪P_{2}^{in}+\omega_{2}{}^{\prime }▪P_{2}^{tmp}+\omega_{3}{}^{\prime }▪Resize\left(P_{1}^{out}\right)}{\omega_{1}{}^{\prime }+\omega_{2}{}^{\prime }+\omega_{3}{}^{\prime }+\beta }\right)$$

where ω_*i*_ is the adaptive weight that represents the contribution of each input, and is set from 0 to 1; β = 0.0001 is to avoid numerical instability; $P_{\text{2}}^{tmp}$ is the intermediate feature, and $P_{2}^{out}$ is the final output at this level. To improve the fusion effect, batch normalization and Mish activation were applied after each convolution.

Moreover, CRB-Net uses the following schemes: CutMix (Yun et al., 2019), DropBlock regularization (Ghiasi et al., 2018), CIoU-loss (Zheng et al., 2020), CmBN (Yao et al., 2020), and NMS (Bodla et al., 2017). We tried to use Mosaic data augmentation (Bochkovskiy et al., 2020), class label smoothing (Szegedy et al., 2016), and cosine annealing scheduler (Loshchilov and Hutter, 2016), but these schemes did not work well.

## Experiment and Discussion

The purpose of object detection is to accurately recognize the categories and position information of various objects in the image.

### Benchmark Testing Methods

Compared with traditional object detection methods, the object detection methods based on deep learning are attracting increasing attention in academia because of their excellent performance in general object detection.

Excluding CRB-Net, two traditional object detection methods and 14 methods based on deep learning were selected in this paper to carry out the baseline test for the dataset. Notably, Yolov3-2SMA and ShipYolo are two methods specifically designed for surface target detection. It is Table 3 lists these 16 state-of-the-art object detection methods and their backbones.

**Table 3:** Sixteen benchmark testing methods and their backbones.

**Method**	**Backbone**
DPM (Felzenszwalb et al., 2010)	/
RANSAC-SVM (Debnath et al., 2015)	/
Faster R-CNN (Ren et al., 2016)	VGG-16
Mask R-CNN (He et al., 2017)	ResNet-101
Cascade R-CNN (Cai and Vasconcelos, 2018)	ResNet-101
TridentNet (Li et al., 2019)	ResNet-101-DCN
SSD (Liu et al., 2016)	VGG-16
RetinaNet (Lin et al., 2017)	ResNet-50
Yolov3 (Redmon and Farhadi, 2018)	Darknet-53
RFBNet (Liu et al., 2018)	VGG-16
M2Det (Zhao et al., 2019)	VGG-16
CenterNet (Duan et al., 2019)	ResNet-50
EfficientDet (Tan et al., 2020)	EfficientNet
Yolov4 (Bochkovskiy et al., 2020)	CSPDarknet-53
Yolov3-2SMA (Li et al., 2020)	Darknet-53
ShipYolo (Han et al., 2021)	CSPDarknet-53

### Evaluation Indexes

A true positive (TP) is defined as the number of detection boxes in which the model correctly predicts the positive class. A true negative (TN) represents the number of detection boxes in which the model correctly predicts the negative class. A false positive (FP) is defined as the number of detection boxes in which the model incorrectly predicts the positive class. A false negative (FN) represents the number of detection boxes in which the model incorrectly predicts the negative class.

The results of the detectors were evaluated by using metrics such as FPS, Intersection over Union (IoU), average precision (AP), and mean average precision (mAP).

Frames per second represents the number of images detected by the method per second.

AP_50_ represents the AP when IoU is 0.5. If IoU exceeds 0.5, the detection will be considered successful and it will be recorded as a TP. If IoU is <0.5, it will be considered as false alarm and recorded as an FP. Undetected will be denoted as an FN.

Intersection over Union involves dividing the area of overlap by the area of union. When IoU exceeds 0.5, the detection is considered successful and it is recorded as a TP.


$$\begin{array}{r} IoU=\frac{\text{Detection Result}\cap \text{Ground Truth}}{\text{Detection Result}\cup \text{Ground Truth}} \end{array}$$

(3) $$\begin{array}{r} =\frac{TP}{TP+FP+FN} \end{array}$$

Average precision is the ratio of precision rate to recall rate on the precision-recall curve. The larger the value, the better the detection effect of the classifier for a certain category.


(4) $$\begin{array}{r} AP=\overset{1}{\underset{0}{\sum }}\left(r_{n+1}-r_{n}\right)P_{interp}\left(r_{n}+1\right) \end{array}$$

where the calculation method of *P*_*interp*_(*r*_*n*+1_) is shown in Equation (5).


(5) $$\begin{array}{r} P_{interp}\left(r_{n+1}\right)=\max_{\bar{r}:\bar{r}\geq {r_{n}}_{+1}}P \end{array}$$

where *P* represents the highest precision under recall rate. The calculation methods of *P* (precision) and *R* (recall rate) are shown in Equations (6) and (7).


(6) $$\begin{array}{r} P=\frac{TP}{TP+FP} \end{array}$$

(7) $$\begin{array}{r} R=\frac{TP}{TP+FN} \end{array}$$

Here, mAP is the mean value of the average accuracy rate of all of the categories. It measures the detection effect of the classifier on all categories.

### Implementation Details

The operating system of the experimental platform is Ubuntu 16.04, with 80 GB of memory. The GPU used for deep learning is the Nvidia Titan-RTX. The other software packages include Python v3.6.10, Torch v1.2.0, and Torchvision v0.4.0.

The parameter settings of DPM and RANSAC-SVM are identical to Felzenszwalb et al. (2010) and Debnath et al. (2015), respectively. The hyperparameters of CRB-Net are set to the same as (Bochkovskiy et al., 2020). For the other deep learning methods, we set the learning rating at 0.00001, the momentum at 0.90, and the weight decay at 0.0005. Due to the limitations of the GPU, we set the batch size of TridentNet at 2, the batch size of Cascade R-CNN at 4, the batch size of Faster-RCNN at 8, and the batch size of others at 16. Moreover, the other hyperparameters were set to exactly the same as those of the original paper of these methods. During the experiment, the original images were resized to 512 ^*^ 512.

To make a fair comparison between the different detectors, in addition to the detectors based on traditional machine learning (DPM and RANSAC-SVM), the other 15 deep learning-based detectors all use the following schemes: CutMix, DropBlock regularization, CIoU-loss, CmBN, and NMS. To ensure that the distributions of training data and testing data match approximately, we randomly selected 70% of the original images as the training set and 30% as the testing set. It should be noted that in the experiment, we used annotations in the same format as the PSCAL VOC dataset.

### Experimental Results

**Figure 9:**
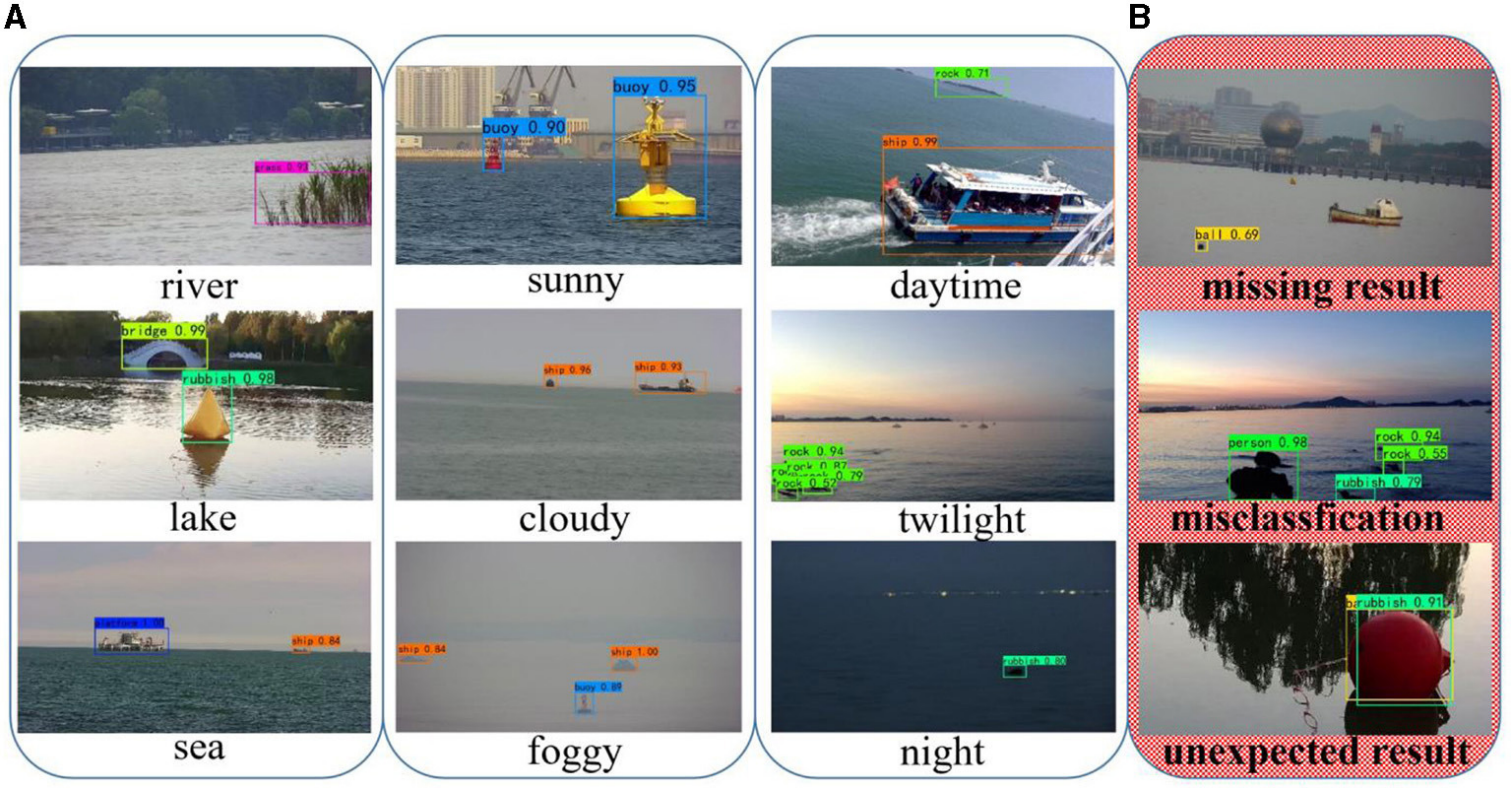
Detection results of CRB-Net. **(A)** Good detection effect (different environments, weather conditions and shooting times). **(B)** Poor detection effect.

Figure 9 shows the detection effect of CRB-Net on some images in WSODD. We selected the detection results in different scenarios, including weather conditions, shooting times, and environments. Figure 9A shows the effectiveness of CRB-Net in different scenarios, while Figure 9B shows areas in which our detector needs further improvement.

The benchmark testing results on WSODD are listed in Table 4 (IoU = 0.5). Columns 3–14 in Table 4 show the AP_50_ for each category in WSODD. This table reveals that traditional machine learning methods have a poor effect not only in detection precision but also in detection speed. The FPS of one-stage detection methods is much faster than that of two-stage detection methods. Furthermore, CRB-Net has the best detection accuracy of all of the benchmark testing methods and its detection speed is also relatively fast.

**Table 4:** Performance benchmarks of 17 methods on WSODD.

**Method**	**FPS**	**mAP (%)**	**AP**_**50**_													
			**Boat (%)**	**Ship (%)**	**Ball (%)**	**Bridge (%)**	**Rock (%)**	**Person (%)**	**Rubbish (%)**	**Mast (%)**	**Buoy (%)**	**Platform (%)**	**Harbor (%)**	**Tree (%)**	**Grass (%)**	**Animal (%)**
DPM	42.16	21.9	9	28	12	34	17	27	29	14	29	32	40	19	15	2
RANSAC-SVM	43.51	27.1	11	49	6	32	33	29	34	7	41	31	27	36	23	20
Faster R-CNN	19.42	32.3	1	73	19	70	14	13	24	14	29	44	53	50	14	4
Mask R-CNN	17.79	35.7	7	79	18	88	27	16	40	22	28	42	61	46	17	8
Cascade R-CNN	29.56	41.1	6	82	22	91	31	19	42	34	31	37	67	63	38	12
TridentNet	10.16	62.2	51	77	37	93	47	57	48	57	66	71	77	70	58	62
SSD	43.02	41.5	41	78	7	79	28	13	28	20	31	47	64	72	29	45
RetinaNet	33.84	27.9	54	73	12	62	26	18	20	7	17	28	31	26	4	11
Yolov3	45.34	56.1	0	83	25	95	40	59	45	60	56	65	89	71	49	48
RFBNet	44.61	35.7	45	69	6	77	24	12	24	15	35	36	56	25	14	62
M2Det	40.63	39.3	0	73	5	83	22	22	25	28	39	46	74	74	20	39
CenterNet	43.42	53.5	70	85	19	93	44	12	44	20	46	61	82	73	48	53
EfficientDet	30.83	31.3	50	75	14	49	26	20	21	16	30	41	25	58	12	0
Yolov4	46.25	57.2	0	85	39	94	51	61	46	60	60	62	83	65	51	45
Yolov3-2SMA	50.46	56.9	0	84	25	92	47	62	46	57	55	69	88	73	44	54
ShipYolo	49.81	58.4	0	87	41	93	52	66	45	63	59	71	78	59	57	56
CRB-Net	**43.76**	**65.0**	**0**	**90**	**69**	**96**	**70**	**71**	**49**	**49**	**59**	**75**	**88**	**72**	**47**	**74**

*The last 14 columns show the AP_50_ for each category on WSODD. The first two methods are traditional machine learning method, the middle four methods belong to two-stage object detection method and the last nine methods are dedicated to one-stage object detection method*.

It can be seen that the detection effect of ball is poor, likely because most of the obstacles in these images are small objects. And the small objects covers fewer pixels, indicating that features used for detection are insufficient and feature representation is weak (Chen et al., 2020a). In addition, the reflection of ball from water surface will also affect the detection result to some extent. In addition, the AP_50_ of boat is low (especially for the Faster RCNN, Yolov3, Yolov4,M2Det, Yolov3-2SMA, ShipYolo, and CRB-Net), possibly because WSODD considers all of the sailing boats, canoes, speedboats and tourist boats as boat, making it difficult for the DNN to extract features of this category. The category of animal cannot be detected very well (especially in Faster RCNN, Mask R-CNN, and EfficientDet), which may be because of the small number of instances of this category. As a result, the fitting effect of the DNN is poor.

To more deeply explore the detection performance for objects of different sizes, we selected nine algorithms with mAP greater than 40% in Table 4 for experiments. During the experiments, an instance whose scale size is less than 10% in its image is marked as a small object, and mAP (small) is obtained when IoU is 0.5. Similarly, mAP (Medium), mAP (Large), and mAP (VeryLarge) represent the AP of medium objects (10–20%), large objects (20% to 30 T), and very large objects (≥30%) (Li et al., 2018). Table 5 shows the test results. It can be seen that, compared to other detectors, CRB-Net has higher precision for the detection of small and medium-sized objects.

**Table 5:** Detection performance for objects of different sizes.

**Method**	**FPS**	**mAP (Small) (%)**	**mAP (Medium) (%)**	**mAP (Large) (%)**	**mAP (VeryLarge) (%)**
Cascade R-CNN	29.51	12.1	17.9	31.9	50.3
TridentNet	9.83	24.9	25.6	49.1	50.8
SSD	43.42	15.6	18.7	28.4	53.7
Yolov3	44.17	23.9	26.2	42.2	56.5
CenterNet	42.98	10.1	24.2	30.3	43.3
Yolov4	45.64	24.2	25.4	42.7	59.2
Yolov3-2SMA	49.86	24.0	25.7	40.2	57.9
ShipYolo	49.27	24.7	25.4	41.9	60.7
CRB-Net	**44.11**	**29.1**	**28.6**	**42.4**	**57.7**

In addition, we experimented with various algorithms using different input sizes. As the input size increased, the detection accuracy became higher. When the input size was in the range of 512 ^*^ 512 pixels to 1,024 ^*^ 1,024 pixels, the detection accuracy of all algorithms improved by more than 3%. The improvement came mainly from small objects, meaning that higher resolution was very useful for detecting small objects. However, detecting objects in higher resolution images has more computational overhead. Therefore, it is necessary to design efficient detectors for high-resolution images in the future.

It is worth noting that the performance of these methods based on this dataset is much lower than that in their original paper. This may be caused by higher image resolution, larger calculation dimension and markedly different object categories. It is for these reasons that this dataset is challenging.

### Discussion

Traditional object detection methods have low detection accuracy and poor real-time performance. Fortunately, the emergence of deep learning has led to a new trend in object detection. It can be concluded from experiments with 15 deep learning benchmark methods that the one-stage object detection method has a big advantage in detection speed, and has also made significant progress in detection precision, which could help to achieve better real-time detection. In the self-driving process of USVs, object detection must have excellent real-time performance in order to meet the information perception and decision-making requirements of USVs. Therefore, the one-stage object detection method will be the mainstream method in this field.

Because the boat category in WSODD contains a variety of boats of different shapes, to prove the poor detection effect of this category, we tried to divide the category of boat into finer detail and used the above-mentioned networks to retrain and redetect. When we did this, we found that the prediction precision was greatly improved. Although the original recognition precision of this category was low, it is still of significance to the detection field. First, the category could be divided in finer detail for further surface object detection studies. Second, it will help to improve the detection effect of categories that contain multiple subcategories with weak correlation. It is obvious that the proposed CRB-NET can significantly improve detection precision while maintaining good detection speed. However, the detection effect of this detector is poor in the detection and recognition of weakly correlated categories. This needs to be further improved.

## Cross-Dataset Validation

Cross-dataset validation is an effective means of evaluating the generalization ability of a dataset and a detector. In this section, a boat-types-recognition dataset is selected to perform cross-dataset generation because it contains a relatively large number of common water surface obstacles.

It should be noted that the boat-types-recognition dataset was annotated manually because it did not provide an annotation file. In this process, the objects in the images of this dataset were divided into nine categories: cruise ship, ferryboat, freight boat, gondola, inflatable boat, kayak, paper boat, sailboat, and buoy. Because there were no official data splits, we randomly selected 70% of the original images as the training set and 30% as the testing set. Seven methods, namely DPM, Faster R-CNN, CenterNet, Yolov4, Yolov3-2SMA, ShipYolo, and CRB-Net, were chosen to evaluate this dataset. In addition, the parameter settings of the methods are exactly the same as those in section Implementation Details.

The results are shown in Table 6. It is important to understand that the boat-types-recognition dataset is mainly composed of three categories of objects: boat (gondola, inflatable boat, kayak, paper boat, sailboat), ship (cruise ship, ferry boat, freight boat), and buoy. Buoy, freight boat, and inflatable boat each have less than 40 images, which is why their detection accuracy is so low. Water Surface Object Detection Dataset contains more categories of objects, more images for each category, and more scenes than the boat-types-recognition dataset.

**Table 6:** Results of cross-dataset generalization.

**Dataset**	**Method**	**FPS**	**mAP (%)**	**AP**_**50**_								
				**Buoy (%)**	**Cruise boat (%)**	**Ferry boat (%)**	**Freight boat (%)**	**Gondola (%)**	**Inflatable boat (%)**	**Kayak (%)**	**Paper boat (%)**	**Sail boat (%)**
Boat-types-recognition	DPM	42.74	38.1	‘17	46	10	15	73	22	39	48	73
	Faster R-CNN	21.14	44.8	31	78	11	0	80	26	41	56	81
	CenterNet	43.44	37.4	11	82	11	4	74	3	52	13	86
	Yolov4	47.46	49.35	14	90	6	8	80	12	66	74	86
	Yolov3-2SMA	49.97	48.11	14	89	3	11	83	7	65	79	82
	ShipYolo	49.56	47.78	12	87	12	6	76	22	68	68	79
	CRB-Net	**44.44**	**53.5**	**8**	**92**	**21**	**8**	**79**	**25**	**76**	**82**	**90**

Moreover, of all the methods, CRB-NET achieves the highest detection accuracy plus a fast detection speed. This proves that the proposed method has outstanding generalization ability and can be applied to different datasets.

## Conclusion

To better evaluate different methods, a high-quality dataset is needed for water surface objective detection. In this paper, an annotated dataset called WSODD is proposed. As the largest image-based dataset, WSODD significantly enhances water surface object detection. In addition, WSODD is a benchmark dataset that contains a variety of water environments, rich lighting conditions, and different weather conditions. It basically covers all of the common obstacles in water environments. The results of 17 object detection methods also provide a standard benchmark for WSODD, which is a solid foundation for other researchers to carry out further work. The results of the experiments prove that the proposed CRB-Net not only ensures good detection speed, but also significantly improves the detection precision, especially for small and medium-sized objects. Finally, cross-dataset validation demonstrates that WSODD would be a pre-eminent dataset, and that CRB-Net has excellent generalization ability.

## Data Availability Statement

The datasets presented in this study can be found in online repositories. The names of the repository/repositories and accession number(s) can be found at: https://github.com/sunjiaen/WSODD; https://github.com/sunjiaen/BTRDA.

## Author Contributions

ZZ and JD initiated and supervised the research. JS wrote the paper and carried out the experiments. JD polished the paper. JS, JY, and KL record the data. LC and CC put forward some effective suggestions for improving the structure of the paper. All authors contributed to the article and approved the submitted version.

## Funding

This work was supported by the Equipment Pre-Research Field Fund Thirteen Five-year (No. 61403120109, BIT) and the Fundamental Research Funds for the Central Universities (No. 21619412, JNU).

## Conflict of Interest

The authors declare that the research was conducted in the absence of any commercial or financial relationships that could be construed as a potential conflict of interest.

## Publisher's Note

All claims expressed in this article are solely those of the authors and do not necessarily represent those of their affiliated organizations, or those of the publisher, the editors and the reviewers. Any product that may be evaluated in this article, or claim that may be made by its manufacturer, is not guaranteed or endorsed by the publisher.
